# Carbapenem resistance in pediatric *Acinetobacter baumannii* urinary tract infections: molecular determinants

**DOI:** 10.1186/s13052-026-02349-9

**Published:** 2026-11-02

**Authors:** Rasha Hassan Hassan, Maysaa El Sayed Zaki, Mohamed Saleh Ismail, Mohamed Ahmed Noureldin, Nourhan Alaa El-Din Youssef

**Affiliations:** 1 Pediatrics- Faculty of Medicine, Mansoura University, Mansoura, Egypt; 2 Clinical Pathology-Faculty of Medicine, Mansoura University, Mansoura, Egypt; 3 Department of Pediatrics, Horus University Faculty of Medicine, Damietta, Egypt

**Keywords:** *Acinetobacter baumannii*, Antimicrobial resistance, Carbapenem resistance, Pediatric UTI, OXA-23, NDM

## Abstract

**Background:**

In the pediatric population, carbapenem-resistant Acinetobacter baumannii (CRAB) is a significant cause of hospital-acquired infections, particularly hospital-acquired urinary tract infections (HA-UTIs). There are a few studies on the molecular mechanisms of resistance to CRAB among children.

**Objective:**

To detect the prevalence of CRAB among children with HA-UTIs and to evaluate associated carbapenemase genes and clinical risk factors.

**Methods:**

This cross-sectional study included 100 children with HA-UTIs. Isolates were identified using standard microbiology techniques, and antimicrobial resistance (AMR) was detected according to the Clinical and Laboratory Standards Institute (CLSI) guidelines. Carbapenemase genes (blaOXA-23-like, blaOXA-24/40-like, blaOXA-58-like, and blaNDM) were detected by PCR. Data were statistically analyzed to assess the association between clinical factors and carbapenem resistance (CR).

**Results:**

CR was identified in 73% of isolates. High rates of AMR were observed for cephalosporins and fluoroquinolones. The most prevalent gene was the blaOXA-23-like gene (60%), followed by the blaOXA-58-like (34%), the blaOXA-24/40-like (30%), and the blaNDM (15%). A significant association was observed between resistance to carbapenems and the carbapenemase genes blaOXA-23-like and blaNDM (*p* < 0.05). Clinical factors like ICU admission and catheterization did not show a significant association with resistance.

**Conclusion:**

Carbapenem resistance among pediatric *Acinetobacter baumannii* UTIs is highly prevalent and primarily driven by molecular mechanisms, particularly OXA-23 and NDM carbapenemases. This underscores the significance of molecular diagnostics and infection control in pediatric healthcare settings.

**Supplementary Information:**

The online version contains supplementary material available at https://doi.org/10.1186/s13052-026-02349-9.

## Introduction

Urinary tract infection (UTI) is a common bacterial infection in the pediatric population and is associated with high morbidity rates, mainly in hospitalized children and children having underlying comorbid conditions. Although *E. coli* remains the predominant bacterium in community-acquired infections, there has been a remarkable shift towards Gram-negative bacteria in hospital-acquired infections, especially in the intensive care unit (ICU) and in catheterized subjects. *Acinetobacter baumannii* (*A. baumannii*) is one of these emerging bacteria. *A. baumannii* has gained a high attention being able to survive in hospital environments and is related to device-associated infections, such as UTI related to urinary catheters [1].

*A. baumannii* is an opportunistic pathogen associated with a variety of hospital-acquired infections, including chest infections, bloodstream infection, wound infection, and UTI. Due to its persistence in hospital environments and its ability to resist desiccation, the transmission of *A. baumannii* is facilitated in health-care settings, predominantly among high-risk children [2].

The AMR in *A. baumannii* has become a global health issue and CRAB has been identified by the WHO as a priority pathogen. This is because CRAB has limited therapeutic options and is linked to increased mortality [3]. Several studies reported > 50% AMR rates, with some reporting 70–90% in hospitals [4]. Also, CRAB is linked to unfavorable outcomes, such as increased length of hospital stay and high mortality rates [5]. Notably, the mechanism that underlies carbapenem resistance is multifactorial, however, carbapenemase genes are believed to have a key role. The most important of these genes are OXA-type β-lactamases, especially blaOXA-23-like, blaOXA-24/40-like, and blaOXA-58-like. These genes are usually encoded on mobile genetic sequences, facilitating horizontal gene transfer. Other genes that have roles in the CR of *A. baumannii* are metallo-β-lactamases (e.g. blaNDM), which have been widely identified globally. This further complicates the therapeutic approaches [6].

To date, significant gaps exist in the literature regarding CRAB. Most studies have focused on adults or on non-urinary infections, whereas data on pediatric hospital-acquired UTIs remain limited. Furthermore, there is a lack of integrated studies that combine phenotypic antimicrobial resistance patterns with molecular detection of carbapenemase genes in children, especially in low- and middle-income countries [7].

So, our study aimed to detect the prevalence of CRAB among pediatric hospital-acquired UTIs (HA-UTIs) and to characterize the molecular mechanisms of CR by detecting the presence of blaOXA-23-like, blaOXA-24/40-like, blaOXA-58-like, and blaNDM genes. The AMR of isolates were also evaluated to detect the prevalence of multidrug-resistant (MDR) and extensively drug-resistant (XDR) *A. baumannii*. Furthermore, the relationship between CR and clinical variables was also assessed. These included duration of hospitalization, catheterization, and ICU admission. Also, the study assessed the association between the presence of carbapenemase genes and phenotypic resistance profiles.

## Materials and methods

### Study design, setting, and reporting

A prospective cross-sectional study was conducted over 24 months (March 2024 to March 2026) in the pediatric wards and ICUs of Mansoura University Children’s Hospital, Mansoura, Egypt. The study investigated the prevalence of CRAB in pediatric HA-UTIs. The Mansoura Faculty of Medicine Ethical Committee, Egypt, approved the study (code: R.26.05.3728), and written consents were obtained from parents before enrollment. The study was performed in accordance with the Helsinki guidelines. The study was reported in accordance with the STROBE Statement. One non-duplicate isolate was obtained per patient.

### Sample size calculation

Sample size underwent calculation using a single-proportion formula based on an expected prevalence of carbapenem-resistant *A. baumannii* of 60%.

$$n = \frac{(Z^2 \times p(1-p))}{d^2}$$

The minimum sample size was 93 isolates, increasing to at least 100.

### Study population

Children aged ≤ 18 years who developed UTI ≥ 48 h after hospital admission were included.

Isolation and Identification of *A. baumannii*.

Urine specimens were aseptically collected from children with HA-UTIs. In the microbiology laboratory, samples were cultured on Cysteine Lactose Electrolyte Deficient agar and MacConkey agar plates (Oxoid, UK) using calibrated loops that deliver 0.001 mL urine. The plates then underwent aerobic incubation for 18–24 h at 37 °C. The bacterial growth of ≥ 10⁵ CFU/mL was identified as “significant bacteriuria”.

Presumptive identification of *A. baumannii* was based on the morphological characteristics of colonies, gram staining procedure, and conventional biochemical characteristics. Colonies appeared as non-lactose fermenting, smooth and pale on MacConkey agar. The staining procedure revealed Gram-negative coccobacilli. Isolates were non-motile and unable to ferment glucose. Isolates showed negative oxidase reaction but positive catalase and citrate reactions. The API 20NE identification system (bioMérieux, France) was utilized for further confirmation, following the manufacturer’s guidelines.

### Antibiotic susceptibility test

The antibiotic susceptibility test was performed using the disk diffusion test on Mueller–Hinton agar (MHA) (Oxoid, UK). Fresh overnight cultures were used to prepare bacterial suspensions equivalent to 0.5 McFarland turbidity standards. Using sterile cotton swabs, bacterial suspensions were uniformly inoculated onto MHA plates.

The antibiotic discs (Oxoid, UK) tested included: imipenem (10 µg), meropenem (10 µg), ceftazidime (30 µg), cefepime (30 µg), ciprofloxacin (5 µg), levofloxacin (5 µg), gentamicin (10 µg), amikacin (30 µg), and piperacillin–tazobactam (100/10 µg). Plates were aerobically incubated for 16–18 h at 37 °C, after which inhibition zone diameters were measured and interpreted in accordance with CLSI.

Isolates that were resistant to at least one carbapenem antibiotic (imipenem and/or meropenem) were classified as CRAB. MDR was defined as non-susceptibility to at least one agent in ≥ 3 antibiotic classes, while XDR isolates included isolates not susceptible to at least one agent in all antibiotic agents except ≤ 2 antibiotic classes. *E. coli* ATCC 25,922 and *Pseudomonas aeruginosa* ATCC 27,853 were used as control strains.

### Phenotypic detection of carbapenemase production by Double-Disc Synergy Method (DDST)

Carbapenemase-producing bacteria were phenotypically detected using the carbapenem DDST. A bacterial suspension equivalent to 0.5 McFarland standard was inoculated onto MHA plate. On the MHA surface, imipenem and meropenem discs (10 µg each) were placed at a standardized distance from discs containing carbapenemase inhibitors, and the plates were incubated for 18–24 h at 37 °C. Following incubation, enhancement or distortion of the inhibition zone toward the inhibitor-containing disc was considered “positive” for carbapenemase production. Isolates demonstrating positive synergy were considered phenotypically positive for carbapenemase activity.

### PCR detection of carbapenemase genes

Genomic DNA extraction from confirmed Acinetobacter baumannii isolates was performed using QIAamp DNA Mini Kit (Qiagen, Germany). DNA concentration and purity were evaluated spectrophotometrically, and the DNA was stored at − 20 °C until use.

Carbapenemase genes were detected by conventional PCR. The primer sequences used in this study are presented below. Table 1.

**Table 1:** The carbapenamse genes and the sequences of the used primers

Carbapenemase gene	Primer sequences (5′–3′)
blaOXA-23-like	F: GATCGGATTGGAGAACCAGA R: ATTTCTGACCGCATTTCCAT
blaOXA-24/40-like	F: GGTTAGTTGGCCCCCTTAAA R: AGTTGAGCGAAAAGGGGATT
blaOXA-58-like	F: AAGTATTGGGGCTTGTGCTG R: CCCCTCTGCGCTCTACATAC
blaNDM	F: GGTTTGGCGATCTGGTTTTC R: CGGAATGGCTCATCACGATC

PCR was amplified in a final volume of 25 µL as follows: 12.5 µL of PCR Master Mix (Thermo Scientific, USA), 1 µL of each primer, 5 µL of extracted DNA, and nuclease-free water to complete the reaction volume.

A thermal cycler was used for DNA amplification under standard PCR conditions as follows: initial denaturation at 95 °C for 5 min, followed by 35 cycles, each consisting of denaturation at 95 °C for 30 s, annealing at 52–58 °C for 30 s, and extension at 72 °C for 45 s. The final step was extension for 7 min at 72 °C. PCR products were analyzed by electrophoresis on a 1.5% agarose gel stained with ethidium bromide and visualized by UV transillumination. A 100-bp DNA ladder was utilized as a molecular size marker. In all PCR runs, positive and negative controls were included [8, 9].

The detected genes were correlated with AMR and phenotypic carbapenem resistance profiles.

### Statistical analysis

Data will be analyzed using SPSS version 25 (IBM Corp., Armonk, NY, USA). Categorical variables will be presented as frequencies and percentages, whereas continuous variables will be summarized as mean ± standard deviation or median with interquartile range, according to data distribution. The Shapiro–Wilk test will be used to assess the normality of continuous variables.

The primary outcome will be **carbapenem resistance**, defined as resistance to at least one of the tested carbapenems (imipenem and/or meropenem), and will be analyzed as a binary variable (CRAB vs. non-CRAB). Associations between CRAB status and categorical clinical variables, including sex, ICU admission, and urinary catheterization, will be assessed using the chi-square test or Fisher’s exact test, as appropriate. The associations between CRAB status and the detected carbapenemase genes (**blaOXA-23-like**,** blaOXA-24/40-like**,** blaOXA-58-like**,** and blaNDM**) will similarly be evaluated using Fisher’s exact test when expected cell counts are small or sparse.

Because all **blaOXA-23-like-positive** and **blaNDM-positive** isolates were carbapenem-resistant, complete separation was present in conventional maximum-likelihood logistic regression. Therefore, conventional logistic regression will not be used to estimate odds ratios for these separated predictors. Instead, **Firth’s penalized logistic regression** will be used to obtain bias-reduced estimates when complete separation is present. The multivariable model will include sex, ICU admission, urinary catheterization, and the four carbapenemase genes. Firth-adjusted odds ratios (ORs) with **95% profile-likelihood confidence intervals (CIs)** will be reported. Female sex, ward admission, absence of urinary catheterization, and gene-negative status will serve as the respective reference categories.

The results of the univariable exact analyses will be reported as numbers, percentages, and Fisher’s exact *P* values, whereas the multivariable analysis will be reported as Firth-adjusted ORs with 95% CIs and corresponding *P* values. All statistical tests will be two-sided, and a *P* value < 0.05 will be considered statistically significant.

## Results

### Patient characteristics

One hundred children with *A. baumannii*-related HA-UTIs were included in this study. Of the children, there were 52 males and 48 females (52.0% and 48.0%, respectively).

Regarding clinical distribution, 47 children (47.0%) were admitted to ICUs, while 53 children (53.0%) were managed in general pediatric wards. Urinary catheterization was present in 47 patients (47.0%), whereas 53 patients (53.0%) were non-catheterized. The age distribution showed that 33 patients (33.0%) were ≤ 5 years, 32 (32.0%) were 6–10 years, and 35 (35.0%) were 11–15 years. No patients were older than 15 years.

The duration of hospitalization was ≤ 1 week in 27 children (27.0%), 1–2 weeks in 28 children (28.0%), and > 2 weeks in 45 children (45.0%). Table 2.

### Antimicrobial susceptibility and co-resistance patterns

Antimicrobial susceptibility analysis demonstrated substantial resistance among the 100 *A. baumannii* isolates. Meropenem resistance was detected in 73 isolates (73.0%), and imipenem resistance in 56 (56.0%). Isolate-level analysis showed that 56 isolates (56.0%) were resistant to both carbapenems, while 17 (17.0%) were resistant to meropenem but susceptible to imipenem. No isolate demonstrated imipenem resistance in the absence of meropenem resistance, and 27 isolates (27.0%) were susceptible to both agents. Consequently, the 73 isolates resistant to either imipenem or meropenem were classified as CRAB according to the predefined study criteria.

Resistance to the other antimicrobial agents varied considerably. The highest non-carbapenem resistance rates were observed for ciprofloxacin (73.0%), ceftazidime (69.0%), and cefepime (67.0%), followed by gentamicin (55.0%). Resistance to levofloxacin, amikacin, and piperacillin–tazobactam was comparatively lower, at 42.0%, 45.0%, and 43.0%, respectively.

When resistance was examined specifically among CRAB isolates, all isolates were meropenem-resistant (73/73, 100.0%), and 56/73 (76.7%) were imipenem-resistant. Among the non-carbapenem agents, ciprofloxacin showed the highest rate of resistance among CRAB isolates (55/73, 75.3%), followed by ceftazidime (52/73, 71.2%) and cefepime (50/73, 68.5%). Resistance to gentamicin was detected in 38/73 (52.1%) CRAB isolates, whereas amikacin and piperacillin–tazobactam resistance occurred in 30/73 (41.1%) and 29/73 (39.7%), respectively. None of the non-carbapenem resistance rates differed significantly between CRAB and non-CRAB isolates (all *P* > 0.05), although levofloxacin resistance showed a trend toward being more frequent among CRAB isolates (47.9% vs. 25.9%, *P* = 0.067).

The pairwise co-resistance analysis demonstrated that carbapenem resistance frequently co-occurred with resistance to other antimicrobial classes. Among CRAB isolates, the most frequent co-resistance patterns involved meropenem with imipenem (56/73, 76.7%), ciprofloxacin (55/73, 75.3%), ceftazidime (52/73, 71.2%), and cefepime (50/73, 68.5%). Meropenem resistance also co-occurred with gentamicin in 38 (52.1%), levofloxacin in 35 (47.9%), amikacin in 30 (41.1%), and piperacillin–tazobactam in 29 (39.7%) CRAB isolates.

Overall, these findings demonstrate that although resistance to amikacin and piperacillin–tazobactam was lower than resistance to carbapenems and several other agents, these antibiotics were nevertheless frequently involved in co-resistance patterns among CRAB isolates. The isolate-level analysis, therefore, provides a more detailed representation of the antimicrobial resistance profile than the aggregate resistance percentages alone. Table 3.

### Distribution of carbapenemase genes

The blaOXA-23-like, blaOXA-24/40-like, blaOXA-58-like, and blaNDM genes were detected in 60 (60%), 30 (30%), 34 (34%), and 15 isolates (15%), respectively. Table 4.

Isolate-level analysis demonstrated frequent co-carriage of the investigated carbapenemase genes. Multiple carbapenemase genes were detected in 45/100 isolates (45.0%), including two genes in 38 isolates (38.0%) and three genes in 7 isolates (7.0%); no isolate carried all four investigated genes. A single carbapenemase gene was detected in 42 isolates (42.0%), while 13 isolates (13.0%) were negative for all four targets. The most frequent two-gene combinations were *bla*OXA-23-like + *bla*OXA-24/40-like and *bla*OXA-23-like + *bla*OXA-58-like, each occurring in 12 isolates (12.0%), followed by *bla*OXA-23-like + *bla*NDM in 9 isolates (9.0%). Three-gene combinations were identified in 7 isolates (7.0%). Tables 5, 6.

### Relationship between clinical variables and CRAB

Carbapenem resistance was observed in 34 of 48 female patients (70.83%) and 39 of 52 male patients (75.0%) (*p* = 0.8076).

Among ICU patients, resistance was detected in 33 of 47 cases (70.21%), compared to 40 of 53 ward patients (75.47%) (*p* = 0.7147).

Among catheterized patients, resistance was detected in 32 of 47 cases (68.09%), compared to 41 of 53 non-catheterized patients (77.36%) (*p* = 0.414). Table 7.

### Carbapenemase gene associations and multivariable analysis

All isolates harboring *bla*OXA-23-like were carbapenem-resistant (60/60, 100.0%), compared with 13/40 (32.5%) among isolates without the gene (*P* < 0.001). Similarly, all *bla*NDM-positive isolates were carbapenem-resistant (15/15, 100.0%), compared with 58/85 (68.2%) among *bla*NDM-negative isolates (*P* = 0.009). No significant associations were observed for *bla*OXA-24/40-like (*P* = 0.461) or *bla*OXA-58-like (*P* = 0.096). These findings indicate that, among the carbapenemase genes investigated, *bla*OXA-23-like showed the strongest association with the CRAB phenotype. Table 8.

Because complete separation occurred for *bla*OXA-23-like and *bla*NDM in conventional logistic regression, Firth penalized logistic regression was used for multivariable analysis. *bla*OXA-23-like remained independently associated with carbapenem resistance (OR, 169.52; 95% CI, 22.81–21,276.50; *P* < 0.001). *bla*NDM showed an elevated but statistically non-significant association after adjustment (OR, 11.19; 95% CI, 0.94–1,526.65; *P* = 0.056). Neither *bla*OXA-24/40-like nor *bla*OXA-58-like was independently associated with CRAB. Likewise, sex, ICU admission, and urinary catheterization were not significant independent predictors of carbapenem resistance (all *P* > 0.05) Tables 9 and 10.

Among the clinical variables evaluated, no significant association with CRAB status was observed for sex, ICU admission, or urinary catheterization (all *P* > 0.05). These analyses were restricted to the clinical variables available in the study dataset and therefore do not exclude the potential contribution of other healthcare-associated exposures or patient-level risk factors.

Representative PCR amplification products and corresponding positive and negative controls are provided in Supplementary Figure S1.

**Table 2:** Demographic and clinical characteristics (No. and %)

Variable	Category	No.	Percentage (%)
Sex	F	48	48.0
Sex	M	52	52.0
Unit	ICU	47	47.0
Unit	Ward	53	53.0
Catheter	No	53	53.0
Catheter	Yes	47	47.0
Age	≤ 5	33	33.0
Age	6–10	32	32.0
Age	11–15	35	35.0
Age	> 15	0	0.0
Hospital_Days	≤ 7	27	27.0
Hospital_Days	8–14	28	28.0
Hospital_Days	> 14	45	45.0

**Table 3:** Cumulative antimicrobial resistance profile of A. baumannii isolates

Antimicrobial / phenotype	Resistant, *n* (%)	95% CI
Imipenem	56 (56.0)	45.7–65.9
Meropenem	73 (73.0)	63.2–81.4
Ceftazidime	69 (69.0)	59.0–77.9
Cefepime	67 (67.0)	56.9–76.1
Gentamicin	55 (55.0)	44.7–65.0
Amikacin	45 (45.0)	35.0–55.3
Ciprofloxacin	73 (73.0)	63.2–81.4
Levofloxacin	42 (42.0)	32.2–52.3
Piperacillin–tazobactam	43 (43.0)	33.1–53.3
CRAB	73 (73.0)	63.2–81.4
MDR	96 (96.0)	90.1–98.9
XDR	20 (20.0)	12.7–29.2

Values are based on 100 isolates. 95% CIs were calculated using the exact Clopper–Pearson binomial method. CRAB, carbapenem-resistant A. baumannii; MDR, multidrug-resistant; XDR, extensively drug-resistant

**Table 4:** Prevalence of carbapenemase genes among A. baumannii isolates

Carbapenemase gene	Positive, *n* (%)	95% CI
blaOXA-23-like	60 (60.0)	49.7–69.7
blaOXA-24/40-like	30 (30.0)	21.2–40.0
blaOXA-58-like	34 (34.0)	24.8–44.2
blaNDM	15 (15.0)	8.6–23.5

Values are based on 100 isolates. Gene categories are not mutually exclusive. 95% CIs were calculated using the exact Clopper–Pearson binomial method

**Table 5:** Isolate-level distribution and co-carriage patterns of carbapenemase genes among 100 A. baumannii isolates

Carbapenemase genotype	n (%)	95% CI
No carbapenemase gene detected	13 (13.0)	7.1–21.2
Single gene only	42 (42.0)	32.2–52.3
Two genes	38 (38.0)	28.5–48.3
Three genes	7 (7.0)	2.9–13.9
Four genes	0 (0.0)	0.0–3.6
Any multiple-gene carriage (≥2 genes)	45 (45.0)	35.0–55.3
Total	100 (100.0)	—

Percentages are based on all 100 isolates. Multiple-gene carriage was defined as detection of ≥2 of the four investigated carbapenemase genes. 95% CIs were calculated using the exact Clopper–Pearson binomial method

**Table 6:** Detailed carbapenemase gene co-carriage patterns

Gene combination	*n* (%)	95% CI
blaOXA-23-like only	20 (20.0)	12.7–29.2
blaOXA-24/40-like only	8 (8.0)	3.5–15.2
blaOXA-58-like only	12 (12.0)	6.4–19.9
blaNDM only	2 (2.0)	0.2–7.0
blaOXA-23-like + blaOXA-24/40-like	12 (12.0)	6.4–19.9
blaOXA-23-like + blaOXA-58-like	12 (12.0)	6.4–19.9
blaOXA-23-like + blaNDM	9 (9.0)	4.2–16.4
blaOXA-24/40-like + blaOXA-58-like	4 (4.0)	1.1–10.0
blaOXA-24/40-like + blaNDM	1 (1.0)	0.0–5.4
blaOXA-58-like + blaNDM	0 (0.0)	0.0–3.6
blaOXA-23-like + blaOXA-24/40-like + blaOXA-58-like	4 (4.0)	1.1–10.0
blaOXA-23-like + blaOXA-58-like + blaNDM	2 (2.0)	0.2–7.0
blaOXA-23-like + blaOXA-24/40-like + blaNDM	1 (1.0)	0.0–5.4
Four-gene carriage	0 (0.0)	0.0–3.6

Percentages are based on all 100 isolates. Categories are mutually exclusive. 95% CIs were calculated using the exact Clopper–Pearson binomial method

**Table 7:** Risk factors associated with CRAB

Variable	Category	Total	CRAB_Positive	%	p_value
Sex	F	48	34	70.83	0.8076
Sex	M	52	39	75.0	0.8076
Unit	ICU	47	33	70.21	0.7147
Unit	Ward	53	40	75.47	0.7147
Catheter	No	53	41	77.36	0.414
Catheter	Yes	47	32	68.09	0.414

**Table 8:** Association between Genes and CRAB

Gene	Status	Total	CRAB_Positive	%	p_value
blaOXA23	Negative	40	13	32.5	0.0
blaOXA23	Positive	60	60	100.0	0.0
blaOXA24	Negative	70	53	75.71	0.4914
blaOXA24	Positive	30	20	66.67	0.4914
blaOXA58	Negative	66	52	78.79	0.1144
blaOXA58	Positive	34	21	61.76	0.1144
blaNDM	Negative	85	58	68.24	0.0251
blaNDM	Positive	15	15	100.0	0.0251

**Table 9:** Exact associations between carbapenemase genes and CRAB status

Gene	CRAB among gene-positive, *n*/*N* (%)	CRAB among gene-negative, *n*/*N* (%)	Fisher’s exact *P*
blaOXA-23-like	60/60 (100.0%)	13/40 (32.5%)	< 0.001
blaOXA-24/40-like	20/30 (66.7%)	53/70 (75.7%)	0.461
blaOXA-58-like	21/34 (61.8%)	52/66 (78.8%)	0.096
blaNDM	15/15 (100.0%)	58/85 (68.2%)	0.009

CRAB, carbapenem-resistant A. baumannii. Fisher's exact test was used because of sparse cells and complete separation for blaOXA-23-like and blaNDM

**Table 10:** Firth penalized multivariable logistic regression for carbapenem resistance

Predictor	Firth-adjusted OR	95% CI lower	95% CI upper	Profile-likelihood *P*
Male	1.28	0.31	5.35	0.733
ICU	0.83	0.20	3.30	0.784
Catheter	0.72	0.17	2.92	0.638
blaOXA-23-like	169.52	22.81	21276.50	< 0.001
blaOXA-24/40-like	0.49	0.08	2.19	0.359
blaOXA-58-like	0.51	0.10	2.10	0.353
blaNDM	11.19	0.94	1526.65	0.056

Dependent variable: CRAB (yes/no). Reference categories: female sex, ward admission, no urinary catheterization, and gene-negative status. ORs are Firth-penalized estimates; 95% CIs are profile-likelihood CIs. P values are likelihood-ratio profile tests

## Discussion

The current study integrated clinical, microbiologic, and molecular findings to comprehensively evaluate CRAB in pediatric HA-UTIs. Our results indicate that carbapenem resistance is primarily linked to genetic factors rather than clinical characteristics.

The demographic data demonstrated a balanced sex distribution and comparable ICU and ward admissions. This agrees with other reports indicating that infections caused by *A. baumannii* are primarily associated with the healthcare environment rather than with clinical variables such as sex [10]. The high proportion of long hospital stays further indicates that prolonged hospitalization is a risk factor for acquiring MDR pathogens [11].

In our study, *A. baumannii* isolates exhibited high rates of resistance to carbapenems, cephalosporins, and fluoroquinolones. The widespread dissemination of CRAB, particularly in hospital settings, is indicated by the high rate of meropenem resistance (73%) in our study, consistent with global reports [12]. Also, increased resistance to fluoroquinolones indicates selective pressure from the use of broad-spectrum antimicrobial agents [13, 14].

The antimicrobial susceptibility profile observed in the present study should be interpreted in the context of the substantial geographic and institutional heterogeneity reported for CRAB. CRAB has disseminated globally and is recognized as a critical-priority antimicrobial-resistant pathogen because of its extensive resistance and limited therapeutic options. In the current cohort, meropenem resistance (73.0%) exceeded imipenem resistance (56.0%). Isolate-level analysis showed that 56 isolates were resistant to both carbapenems, whereas 17 were resistant to meropenem but susceptible to imipenem. This pattern, therefore, reflects differential phenotypic susceptibility within the study population rather than a discrepancy in the dataset. Differences in carbapenem susceptibility between agents have been described in *A. baumannii* and may reflect the heterogeneous resistance mechanisms and susceptibility distributions within individual hospital populations; however, the specific mechanism responsible for the observed pattern could not be established in the present study.

The relatively lower resistance observed for amikacin (45.0%) and piperacillin–tazobactam (43.0%) compared with meropenem, ceftazidime, cefepime, and ciprofloxacin should similarly be regarded as a characteristic of the isolates investigated rather than as a general feature of CRAB. The current findings may reflect local antimicrobial selection pressure and the specific population of *A. baumannii* circulating in the pediatric hospital setting. Accordingly, direct comparison of individual resistance percentages with reports from other institutions should be made cautiously because CRAB susceptibility profiles vary by geographic region, healthcare setting, patient population, antimicrobial exposure, and circulating clones. Recent epidemiological evidence emphasizes the widespread dissemination of CRAB, particularly OXA-23-associated lineages, while also demonstrating substantial regional heterogeneity.

An important epidemiological consideration is the potential for persistence and in-hospital transmission of resistant *Acinetobacter* populations. Tozluyurt [15] investigated 82 *Acinetobacter calcoaceticus–baumannii* complex isolates recovered in a Turkish university hospital and reported substantial multidrug resistance, including 73.2% XDR and 12.2% PDR isolates. Among isolates with reduced chlorhexidine susceptibility, pulsed-field gel electrophoresis identified seven distinct clones, providing evidence of vertical transmission within the hospital. These findings are relevant to the present study because 73% of our pediatric *A. baumannii* isolates were CRAB, and resistance to several non-carbapenem agents was also substantial, including ciprofloxacin (73%), ceftazidime (69%), and cefepime (67%). However, the present study was a single-center cross-sectional investigation and analyzed one non-duplicate isolate per patient; molecular typing and environmental sampling were not performed. Therefore, we cannot determine whether the high CRAB prevalence reflects dissemination of hospital-adapted clones, repeated independent acquisition, or a combination of both. The Turkish findings highlight the importance of integrating antimicrobial susceptibility surveillance with molecular epidemiological typing and assessment of potential environmental reservoirs when investigating healthcare-associated *Acinetobacter* transmission.

**Molecular characterization identified*bla*OXA-23-like as the most prevalent carbapenemase determinant in the present pediatric cohort**,** detected in 60.0% of isolates (95% CI**,** 49.7–69.7%)**,** followed by*bla*OXA-58-like (34.0%)**, ***bla*OXA-24/40-like (30.0%)**,** and*bla*NDM (15.0%; 95% CI**,** 8.6–23.5%). These findings are broadly consistent with pediatric literature**,** although substantial regional variation exists. A 2024 systematic review of 24 pediatric studies comprising 1**,**129 cases reported an overall prevalence of 64% for*bla*OXA-23-like and 10.9% for*bla*NDM**, **with the Eastern Mediterranean region showing the highest pooled prevalence of carbapenem resistance** (**88.3%**) [16]. **A pediatric study from Tehran, Iran** [17], **conducted during 2019–2020, reported a considerably higher*prevalence of blaOXA-23-like (93.3%)*among 60*A. baumannii*isolates**,** while*bla*NDM was not detected. In contrast**,** prospective surveillance of hospitalized children in Turkey** [18] **identified*bla*NDM in 31% of carbapenem-resistant*A. baumannii*isolates**,** making NDM the second most frequent carbapenemase after OXA-23; notably**,** all NDM-positive cases occurred in neonates admitted to the NICU** [18]. **Thus**,** the 60% OXA-23 prevalence observed in our study is close to the pooled pediatric estimate**,** whereas the 15% NDM prevalence is modestly higher than the pooled pediatric estimate but substantially lower than that reported in the Turkish neonatal population. Such differences likely reflect variation in age distribution**,** infection versus colonization**,** specimen types**,** healthcare setting**,** antimicrobial exposure**,** and circulating clones. Importantly**,** these comparisons should not be interpreted as evidence of a uniform regional epidemiology**,** but rather as evidence of considerable geographic and institutional heterogeneity in the distribution of carbapenemase determinants**.

The blaNDM gene was identified in only 15% of isolates. This agrees with other studies indicating that metallo-β-lactamases are less prevalent but clinically significant, as they are associated with XDR bacteria [19].

In our study, no significant relationship existed between carbapenem resistance and clinical factors. This suggests that infections caused by *A. baumannii* are primarily related to the healthcare environment and horizontal gene transfer rather than clinical variables [20].

The absence of significant associations between CRAB status and the clinical variables evaluated in this study should be interpreted cautiously. Previous antimicrobial exposure, duration and type of urinary catheterization, prior hospitalization, prior ICU stay, underlying diseases, invasive procedures, and other healthcare-associated exposures are recognized as potentially relevant determinants of acquisition of antimicrobial-resistant A. baumannii. These variables were not sufficiently available for reliable analysis in the present dataset and therefore could not be incorporated into the multivariable model. Consequently, residual confounding cannot be excluded, and the lack of statistically significant associations for sex, ICU admission, or catheterization should not be interpreted as evidence that clinical exposures are unimportant. Prospective studies incorporating detailed longitudinal exposure data are warranted to identify independent clinical risk factors for CRAB acquisition.

However, a significant association was observed between phenotypic resistance and carbapenemase genes. The blaOXA-23-like gene showed 100% relationship with carbapenem resistance. The blaNDM gene was also significantly associated with carbapenem resistance. These results agree with previous reports [21].

Overall, our findings demonstrate strong associations between CRAB and selected carbapenemase determinants, particularly *the blaOXA-23-like determinant*. However, because the present molecular analysis evaluated only four carbapenemase genes and did not investigate other recognized resistance mechanisms, these findings should not be interpreted as indicating that carbapenem resistance is exclusively or primarily determined by the genes investigated. Rather, the results suggest that the detected carbapenemase determinants, particularly *bla*OXA-23-like, may contribute substantially to the carbapenem-resistant phenotype in this study population.

Genetic factors were further confirmed to dominate clinical variables in the multivariate analysis (Table 8). The blaOXA-23-like and blaNDM genes were strongly associated with carbapenem resistance. This agrees with previous studies that investigated highly predictive resistance genes [22].

In the present study, *bla*OXA-23-like was strongly associated with the CRAB phenotype and remained significantly associated after Firth penalized multivariable adjustment. However, this association should not be interpreted as evidence of causality. Detection of a resistance gene by PCR establishes its presence and a statistical association with the phenotype, but does not, by itself, demonstrate that the gene is functionally responsible for the observed resistance. Furthermore, the present molecular assessment did not evaluate other recognized mechanisms of carbapenem resistance, including ISAba1-associated expression changes, porin alterations, efflux pump activity, or additional resistance determinants. Therefore, the contribution of *bla*OXA-23-like and other unmeasured mechanisms to the CRAB phenotype requires further investigation using comprehensive molecular and functional approaches.

### Strengths

Our study has many strengths. First, it integrated clinical, microbiological, and molecular data to comprehensively study CRAB in pediatric HA-UTIs. Another strength is that the study included both phenotypic antibiotic susceptibility testing and molecular identification of carbapenemase genes. This enabled the association between genotype and phenotype to be assessed and validated the results. Third, the pediatric population was studied. This population remains underrepresented in AMR studies, particularly in HA-UTIs. Moreover, we use multivariate logistic regression analysis to evaluate independent predictors of carbapenem resistance. This strengthened our results.

### Limitations of the study

This study also has some limitations. First, it is a single-center study, which may lack the generalizability of its results. Second, the sample size was relatively small, which may reduce the power to detect weaker correlations. Third, the complete separation in multivariate analysis, especially for blaOXA-23-like and blaNDM genes, prevented reliable estimation of adjusted ODs. Also, a few carbapenem genes were evaluated. Of note, we did not evaluate other resistance mechanisms, such as efflux pumps, porin alterations, and additional β-lactamases. Finally, there was no longitudinal follow-up. This limited the capacity to evaluate outcomes and response to therapy. Moreover, molecular characterization was restricted to four selected carbapenemase genes (*bla*OXA-23-like, *bla*OXA-24/40-like, *bla*OXA-58-like, and *bla*NDM). Other potentially important mechanisms of carbapenem resistance, including ISAba1-associated β-lactamase overexpression, efflux pump-mediated resistance, porin alterations, and additional acquired or intrinsic determinants of resistance, were not investigated. Consequently, the contribution of the detected genes to the overall carbapenem-resistant phenotype cannot be considered comprehensive. Another limitation is the restricted molecular characterization of carbapenem resistance. Only four carbapenemase genes—*bla*OXA-23-like, *bla*OXA-24/40-like, *bla*OXA-58-like, and *bla*NDM—were investigated. Other mechanisms that may contribute to carbapenem resistance in *A. baumannii*, including ISAba1-associated overexpression, outer-membrane porin alterations, efflux pump-mediated resistance, and additional carbapenemase or β-lactamase determinants, were not assessed. Consequently, the contribution of the detected genes to the overall carbapenem-resistant phenotype cannot be considered comprehensive. The clinical risk-factor analysis was limited by the availability of clinical exposure variables. Detailed information on previous antibiotic exposure, catheterization duration, prior hospitalization, ICU stay, underlying diseases, invasive procedures, and other healthcare-associated exposures were not sufficiently available for robust analysis. Consequently, residual confounding cannot be excluded, and the observed lack of association between the assessed clinical variables and CRAB should be interpreted with caution.

## Conclusion

This study demonstrated a high prevalence of CRAB among children with hospital-acquired UTIs. Among the carbapenemase determinants investigated, *bla*OXA-23-like showed the strongest association with carbapenem resistance, while *bla*NDM was also associated with the CRAB phenotype in unadjusted analysis. However, the molecular assessment was limited to four selected carbapenemase genes and did not evaluate other resistance mechanisms. Therefore, these findings should be interpreted as associations with selected molecular determinants rather than as evidence that carbapenem resistance is exclusively or predominantly gene-driven. Further studies incorporating comprehensive molecular characterization are warranted to clarify the contribution of additional resistance mechanisms.

These findings highlight the importance of molecular diagnostics, antimicrobial stewardship, and targeted infection control strategies. Multicenter studies with larger sample sizes and expanded molecular analyses are therefore required to elucidate resistance mechanisms further and guide effective therapies in children.

## Supplementary Information

Below is the link to the electronic supplementary material.

Supplementary Material 1

## Data Availability

Data of this study are available upon request from the authors.

## References

[1] Peleg AY, Seifert H, Paterson DL. *Acinetobacter baumannii*: emergence of a successful pathogen. Clin Microbiol Rev. 2008;21(3):538–82. 10.1128/CMR.00058-07 PMC2493088 18625687

[2] Doi Y, Murray GL, Peleg AY. *Acinetobacter baumannii*: evolution of antimicrobial resistance—treatment options. Semin Respir Crit Care Med. 2015;36(1):85–98. 10.1055/s-0034-1398388 PMC4465586 25643273

[3] World Health Organization. Global priority list of antibiotic-resistant bacteria to guide research, discovery, and development of new antibiotics. Geneva: WHO; 2017.

[4] Howard A, O’Donoghue M, Feeney A, Sleator RD. *Acinetobacter baumannii*: an emerging opportunistic pathogen. Virulence. 2012;3(3):243–50. 10.4161/viru.19700 PMC3442836 22546906

[5] Lemos EV, de la Hoz FP, Alvis N, Einarson TR, Quevedo E, Castañeda C, Leon Y, Amado C, Cañon O, Kawai K. Impact of carbapenem resistance on clinical and economic outcomes among patients with Acinetobacter baumannii infection in Colombia. Clin Microbiol Infect. 2014;20(2):174–80. 10.1111/1469-0691.12251. Epub 2013 May 13. PMID: 23668595. 23668595

[6] Evans BA, Amyes SG. OXA β-lactamases. Clin Microbiol Rev. 2014;27(2):241–63. 10.1128/CMR.00117-13 PMC3993105 24696435

[7] Logan LK, Gandra S, Trett A, Weinstein RA, Laxminarayan R. *Acinetobacter baumannii* Resistance Trends in Children in the United States, 1999–2012. J Pediatr Infect Dis Soc. 2019;8(2):136–42. 10.1093/jpids/piy018 PMC6510944 29579216

[8] Woodford N, Ellington MJ, Coelho JM, Turton JF, Ward ME, Brown S, et al. Multiplex PCR for genes encoding prevalent OXA carbapenemases in Acinetobacter spp. Int J Antimicrob Agents. 2006;27(4):351–3. 10.1016/j.ijantimicag.2006.01.004 16564159

[9] Poirel L, Walsh TR, Cuvillier V, Nordmann P. Multiplex PCR for detection of acquired carbapenemase genes. Diagn Microbiol Infect Dis. 2011;70(1):119–23. 10.1016/j.diagmicrobio.2010.12.002 21398074

[10] Logan LK, Renschler JP, Gandra S, Weinstein RA, Laxminarayan R. Carbapenem-resistant *Acinetobacter baumannii* infections in children. J Pediatr Infect Dis Soc. 2019;8(4):311–8.

[11] Miller WR, Arias CA. ESKAPE pathogens: antimicrobial resistance, epidemiology, clinical impact and therapeutics. Nat Rev Microbiol. 2024;22:598–616. 10.1038/s41579-024-01054-w. PMC13147291 38831030

[12] Doi Y, Murray GL, Peleg AY. *Acinetobacter baumannii*: evolution of antimicrobial resistance—treatment options. Semin Respir Crit Care Med. 2015;36(1):85–98. 10.1055/s-0034-1398388 PMC4465586 25643273

[13] Wong D, Nielsen TB, Bonomo RA, Pantapalangkoor P, Luna B, Spellberg B. Clinical and pathophysiological overview of *Acinetobacter* infections. Clin Microbiol Rev. 2017;30(1):409–47. 10.1128/CMR.00058-16 PMC5217799 27974412

[14] Nation RL, Li J. Colistin in the 21st century. Curr Opin Infect Dis. 2009;22(6):535–43. 10.1097/QCO.0b013e328332e672 PMC2869076 19797945

[15] Tozluyurt A. Molecular typing of reduced susceptibility of Acinetobacter calcoaceticus–baumannii complex to chlorhexidine in Turkey by pulsed-field gel electrophoresis. J Med Microbiol. 2024;73(8):001882. 10.1099/jmm.0.001882. 39207836

[16] Chávez RodríguezM, De Mascareñas AH, Vaquera Aparicio DN, et al. Molecular epidemiology of carbapenemase encoding genes in *A. baumannii-calcoaceticus* complex infections in children: a systematic review. JAC Antimicrob Resist. 2024;6(4):dlae098. 10.1093/jacamr/dlae098. PMC11242458 39005591

[17] Yousefi Nojookambari N, Sadredinamin M, Dehbanipour R, Ghalavand Z, Eslami G, Vaezjalali M, Nikmanesh B, Yazdansetad S. Prevalence of β-lactamase-encoding genes and molecular typing of Acinetobacter baumannii isolates carrying carbapenemase OXA-24 in children. Ann Clin Microbiol Antimicrob. 2021;20(1):75. 10.1186/s12941-021-00480-5. PMID: 34702307; PMCID: PMC8549256. PMC8549256 34702307

[18] Karaaslan A, Soysal A, Altinkanat Gelmez G, Kepenekli Kadayifci E, Söyletir G, Bakir M. Molecular characterization and risk factors for carbapenem-resistant Gram-negative bacilli colonization in children: emergence of NDM-producing Acinetobacter baumannii in a newborn intensive care unit in Turkey. J Hosp Infect. 2016;92(1):67–72. Epub 2015 Oct 22. PMID: 26601601. 10.1016/j.jhin.2015.09.011 26601601

[19] Poirel L, Nordmann P. Carbapenem resistance in *Acinetobacter baumannii*: mechanisms and epidemiology. Clin Microbiol Infect. 2006;12(9):826–36. 10.1111/j.1469-0691.2006.01456.x 16882287

[20] Munoz-Price LS, Weinstein RA. *Acinetobacter* infection. N Engl J Med. 2008;358(12):1271–81. 10.1056/NEJMra070741 18354105

[21] Dortet L, Poirel L, Nordmann P. Worldwide dissemination of carbapenemase-producing *Acinetobacter baumannii*. Emerg Infect Dis. 2014;20(5):860–3.

[22] Zarrilli R, Pournaras S, Giannouli M, Tsakris A. Global evolution of multidrug-resistant *Acinetobacter baumannii*. Future Microbiol. 2013;8(12):1565–76. 10.1016/j.ijantimicag.2012.09.008 23127486

