# Supplementary material for: Carbapenem resistance in pediatric Acinetobacter baumannii urinary tract infections: molecular determinants
Source: Ital J Pediatr. 2026 Nov 2;52:159. doi: 10.1186/s13052-026-02349-9 (PMC13729496; doi:10.1186/s13052-026-02349-9)
Supplement: Supplementary file 1 [file 13052_2026_2349_MOESM1_ESM.docx]

**Supplementary Figure S1. Representative PCR amplification of carbapenemase genes among** Acinetobacter baumannii **isolates.** Representative agarose gel electrophoresis results showing amplification of **(A)** bla**OXA-23-like (501 bp), (B)** bla**OXA-24/40-like (246 bp), (C)** bla**OXA-58-like (599 bp), and (D)** bla**NDM (621 bp)**. M, 100-bp DNA molecular-weight ladder; lanes 1–5, representative test isolates; PC, positive control; NC, negative control. The expected amplicon sizes are indicated by arrows.
